# Proliferation marker securin identifies favourable outcome in invasive ductal breast cancer

**DOI:** 10.1038/sj.bjc.6604475

**Published:** 2008-07-01

**Authors:** K Talvinen, J Tuikkala, O Nevalainen, A Rantanen, P Hirsimäki, J Sundström, P Kronqvist

**Affiliations:** 1 Department of Pathology, Turku University Hospital, University of Turku, Kiinamyllynkatu 10, FIN-20520 Turku, Finland; 2 Department of Information Technology, University of Turku, FIN-20014 Turku, Finland; 3 Department of Surgical Hospital, Turku University Hospital, PL 28, FIN-20701 Turku, Finland

**Keywords:** securin, breast cancer, cDNA microarray, immunohistochemistry, proliferation, prognosis

## Abstract

We introduce a new proliferation marker, securin (pituitary tumour-transforming 1 (PTTG1)), analysed in invasive ductal breast carcinomas by cDNA microarrays and immunohistochemistry. In cDNA microarray of a total of 4000 probes of genes, securin was revealed with a significant change in expression among the several proliferation-related genes studied. The value of securin as a proliferation marker was verified immunohistochemically (*n*=44) in invasive ductal breast cancer. In follow-up analyses of the sample of patients, the prognostic value of securin was compared with the established markers of breast cancer proliferation, Ki-67 and mitotic activity index (MAI). Our results of a small sample of patients suggest that low securin expression identifies a distinct subgroup of more favourable outcome among patients with high Ki-67 immunoexpression or high MAI. In univariate analysis of Cox's regression, 10-unit increment of securin immunopositivity was associated with a 2.3-fold overall risk of death due to breast cancer and a 7.1-fold risk of death due to breast cancer in the sample of patients stratified according to the cutoff points of 10 and 20% of securin immunopositivity. We suggest that securin immunostaining is a promising and clinically applicable proliferation marker. The finding urges further prognostic studies with a large sample of patients.

In breast tumour biology, deregulated proliferation is one of the most important features predicting malignant behaviour (Desmedt and Sotiriou, 2006). The prognostic power of mitotic activity and traditional proliferation markers, such as standardised mitotic counts and Ki-67, is established in pathological practice. To benefit individual breast cancer patients, the potential of the traditional prognostic features could still be intensified by additional methods (Olivotto *et al*, 1999; Michels *et al*, 2003; Oestreicher *et al*, 2004; Warwick *et al*, 2004; Jalava *et al*, 2006).

We introduce a new proliferation marker, securin (pituitary tumour-transforming 1 (PTTG1)), which was revealed in cDNA microarray analysis with a significant change in expression among several proliferation-related genes studied in invasive ductal breast cancer. Immunohistochemically, the value of securin as a proliferation marker was confirmed among the established proliferation markers of breast cancer. The results emphasise the potential of securin as a prognostic feature to add information in identifying a specific subgroup of more favourable outcome in invasive ductal breast cancer.

## Materials And Methods

### Patient material

The cDNA microarray analysis was performed on 10 patients and immunohistochemical analysis on 2 patient groups, a total of 44 patients, diagnosed in 1996 and 2004 with invasive ductal breast cancer in Turku University Hospital, Turku, Finland (Table 1). The patients were treated with mastectomy with either sentinel lymph node examination or axillary evacuation, and adjuvant treatment with anti-oestrogen, trastuzumab or cytostatic drugs depending on patient's age, hormone receptor status and CISH-confirmed Her2/neu oncogene amplification. All patients participated in regular clinical follow-up during first 5 postoperative years or until the end point of the study. Complete clinical and follow-up data starting from date of diagnosis were available from all patients. Causes of death were verified from autopsy reports, death certificates and patient files from the Finnish Cancer Registry.

### Methods

#### cDNA microarray analysis

In cDNA microarray analysis, special emphasis was placed on the quality of tissue material. Samples were obtained fresh from the operation theatre during surgery, carefully prepared from fat and connective tissue, fresh frozen in liquid nitrogen and stored at −70°C within 30 min after surgical removal. Normal tissue was obtained from benign breast tissue outside the tumour from five patients to prepare a reference pool for cDNA microarray analysis. The diagnosis of all samples was verified from a consecutive histological slide. Microarray included approximately 4000 probes of genes with proven or suspected roles in human cancer (Turku Centre for Biotechnology, University of Turku and Abo Akademi University, Turku, Finland). Total RNA isolation, purification, labelling and microarray hybridisation have been described previously (Talvinen *et al*, 2006). Briefly, 21.5 *μ*g of tumour and reference RNAs was fluorescently labelled (CyDye, Amersham Biosciences, Buckinghamshire, England) with Cy5 and Cy3, respectively, during cDNA synthesis using an oligo(dT) primer (Amersham Biosciences) and Superscript II reverse transcriptase (Gibco BRL Life Technologies, Rockville, MD, USA). Hybridisation was performed in a humidified chamber under LifterSlips (Erie Scientific Company, Portsmouth, NH, USA) overnight at 65°C. Each sample was hybridised once. The sequences of the relevant up- and downregulated genes were verified (Turku Centre for Biotechnology).

#### Immunostainings

Immunostainings of securin and Ki-67 were performed on 4-*μ*m-thick formalin-fixed and paraffin-embedded sections according to a standard procedure. For securin immunostainings, antigen retrieval was performed by repeated microwave heating in 10 mM sodium citrate buffer (pH 6), with monoclonal antibody applied manually at a concentration of 1 : 20 (clone DCS-280, ab3305, Abcam, Cambridge, UK), and detection was performed by biotin-avidin reaction (Vectastain ABC reagent, Vector Laboratories, Burlingame, CA, USA) with diaminobenzidine as chromogen (Sigma, St Louis, MO, USA). Automated immunostaining machine TechMate 500^+^ was used for Ki-67 (clone MIB-1, M 7240, DakoCytomation, Glostrup, Denmark, concentration 1 : 100) with the peroxidase/diaminobenzidine multilink detection kit (DakoCytomation).

#### Mitotic counts

Mitotic counts were determined as mitotic activity index (MAI) (number of mitoses per 10 high-power fields (HPFs), 450 *μ*m in diameter) according to the original publication by Baak *et al* (1985).

#### Evaluation of securin and Ki-67 immunoreactivity, and MAI

Evaluation of securin and Ki-67 immunoreactivity, and MAI was performed on whole carcinoma sections based on the observed and reported (Ogbagabriel *et al*, 2005) focal nature of proliferation in tumour tissue. Securin and Ki-67 immunopositivities were determined by the fraction (%) of positively stained tumour cells and securin also by the intensity of staining (0, no staining; 1, weakly stained; 2, moderately stained; 3, strongly stained) at the areas of most pronounced staining, usually at the most cellular, infiltrating border of the tumour. Analysis of Ki-67 positivity was based on cutoff points at 10 and 20% of cancer cells (Ki-67<10%, 10%⩽Ki-67⩽20% and Ki-67>20%) adopted from clinical practice and based on research on breast cancer samples from Finland and other countries (Kronqvist *et al*, 2004; de Azambuja *et al*, 2007; Railo *et al*, 2007). The same cutoff points were applied for securin immunohistochemistry. Also the cutoff points for MAI were set at 10 and 20 mitoses in 10 HPFs based on the original work of Baak *et al* (1985), the established guidelines of breast cancer grading (Elston and Ellis, 1991; Tavassoli and Devilee, 2003) and our previous results on Finnish patients (Kronqvist *et al*, 2000). The quality of interpretations of immunohistochemistry and MAI was verified as intra- and interobserver reproducibilities in repeated evaluations by a single observer or two independent observers by light microscopy without knowledge of the patients' clinical data.

#### Statistical analysis

Statistical analysis of cDNA microarray experiments was performed as described previously (Talvinen *et al*, 2006). For each sample, the expression ratio between the study case and reference pool was determined for each transcript on the microarray. The expression ratios were log2-transformed and intensity-normalised with the locally weighted scatter plot smoothing (LOWESS; Yang *et al*, 2002). There were three technical replicates for each transcript on the array. The quality of each spot was determined visually and the data were preprocessed by calculating the signal over the whole data so that only spots with acceptable quality were taken into account. When there were three proper spots, the median of the replicates was used to calculate the expression ratio of the transcript. In case of two proper spots, the mean was used, and in case of only one proper spot the value was used as such. If no proper spot was found, then the transcript was marked as missing for the sample under study. Only transcripts with more than 3 non-missing signal values among the 10 samples were subjected to statistical analyses. For each transcript, the preprocessed log-ratios were compared to zero with the one-sample Student's *t*-test. A transcript was considered upregulated if its mean expression ratio was larger than 0.5 and the *P*-value of the *t*-test was less than 0.05. Similarly, a transcript was considered downregulated if its mean expression ratio was less than −0.5 and the *P*-value was less than 0.05. The resulting gene lists were examined for biological information in the context of Gene Ontology by using the GoMiner program package (Zeeberg *et al*, 2003).

Intraobserver (PK) and interobserver reproducibilities of immunohistochemical evaluations of securin and Ki-67 (PK and PH) and evaluation of MAI (PK and JS) described the consistency of repeated assessments by a single observer or a pair of independent observers. They were expressed as intraclass correlation coefficients (ICCs) and weighted kappa coefficients (*κ*_w_) based on cross-tabulations according to the chosen cutoff points. The same statistical analyses were applied for intermethod reproducibilities, and comparisons of the results for securin *vs* MAI, securin *vs* Ki-67 and MAI *vs* Ki-67 were expressed with the help of scatter plots.

Prognostic associations between proliferation markers and survival in breast cancer patients were studied using survival analysis. The cumulative percentages for survival were estimated using the Kaplan–Meier technique and the differences in cumulative percentages between categories were tested using the log-rank test. Proliferation markers were analysed also with the Cox regression model as continuous and categorised variables. Differences were quantified by hazard ratios (HRs) and 95% confidence intervals (95% CIs). The HRs describe the risk of death due to breast cancer associated with values of each studied proliferation marker above the cutoff point as compared with values below the cutoff point. *P*-values less than 0.05 were considered as statistically significant. All statistical computations were performed with SAS statistical package (SAS Institute, release 8.2.2001, Cary, NC, USA).

## Results

**Figure 1:**
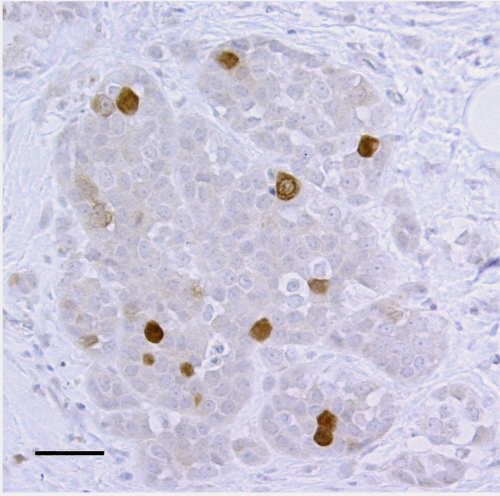
Securin immunohistochemistry in invasive ductal breast cancer. The bar represents 50 *μ*m.

cDNA microarray analysis revealed a total of 131 transcripts upregulated and 256 transcripts downregulated in our sample of invasive breast cancers, corresponding to 119 and 224 up- and downregulated genes, respectively. These represented several significantly deregulated gene groups encoding proteins participating in DNA replication, regulation of cell proliferation, protein biosynthesis, superoxide dismutase activity and cytokine production. We concentrated on proliferation-related genes, among which securin showed clear deregulation and the most promising clinical applications with a consistent immunohistochemical expression pattern (Figure 1).

**Figure 2:**
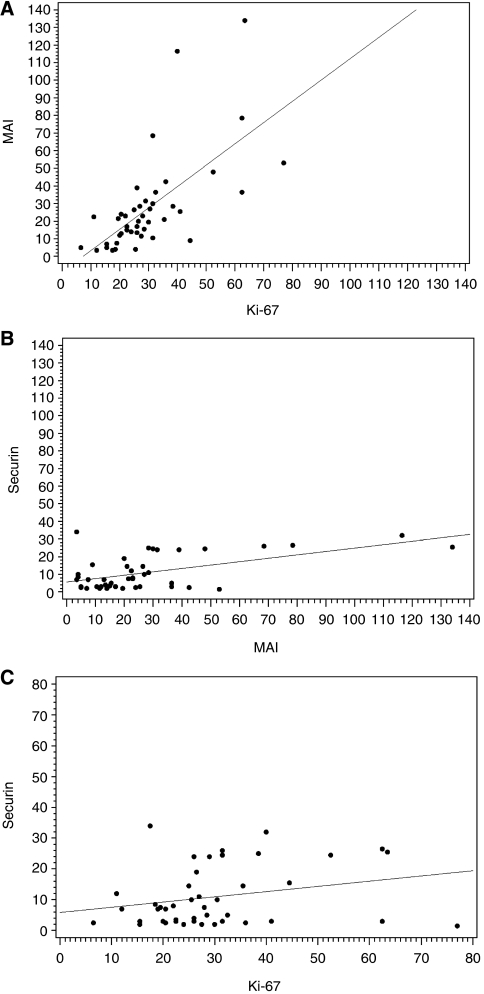
(**A**–**C**) Scatter plots with regression lines demonstrate differences in intermethod consistency between MAI and Ki-67 immunohistochemistry (**A**), securin immunohistochemistry and MAI (**B**), and securin and Ki-67 immunohistochemistry (**C**) for invasive ductal breast cancer cases (*n*=44).

In immunohistochemical analysis, the average fraction of securin-positive cells was 10.9% (range 1.5–34.0) and that of Ki-67-positive cells 32.4% (range 4.0–80.0). Average MAI was 28.0 mitoses per 10 HPFs (range 2–141 mitoses per 10 HPFs). Consistency of evaluations as expressed in intra- and interobserver reproducibilities of each proliferation marker is summarised in Table 2. In our analysis, the intermethod reproducibility between MAI and Ki-67 was moderately high (ICC 0.5527, *κ*_w_ 0.2592; Figure 2A). Interpretations of proliferative activity, however, differed considerably between securin and MAI (ICC 0.1582, *κ*_w_ 0.1899; Figure 2B), and between securin and Ki-67 (ICC −0.2180, *κ*_w_ 0.0619; Figure 2C). In a detailed analysis, securin and Ki-67 resulted in 75% of cases differently allocated into groups of low, intermediate and high proliferation. Securin and MAI, in turn, allocated 64% of cases into different proliferation groups. In our analysis, the lowest consistency between observed proliferation rates was found among cases with low (<10%) securin expression and intermediate or high (⩾10%) Ki-67 expression (57% of cases), and low (<10%) securin expression and intermediate or high (⩾10%) MAI (45% of cases). Including the evaluation of the intensity of securin staining in the analysis did not improve statistical associations.

**Figure 3:**
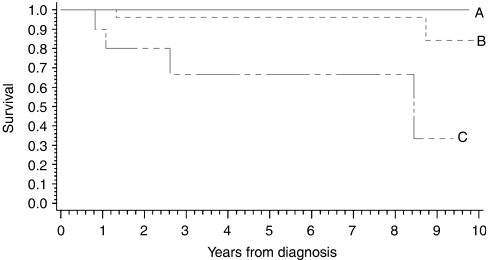
Kaplan–Meier curves of securin immunohistochemistry distinguish patients with low (<10% securin immunopositivity), intermediate (10–20% securin immunopositivity) and high (>20% securin immunopositivity) risk of death due to breast cancer (*n*=44) (*P*=0.0112).

The results of univariate analysis of Cox's regression with HRs of overall risk of death due to breast cancer are summarised in Table 3 and show statistically significant prognostic value for securin immunohistochemistry and MAI (HRs 2.3 and 1.3, respectively). In our sample, only securin immunohistochemistry demonstrated statistically significant prognostic stratification of patients according to cutoff points of 10 and 20% of breast cancer cells. Among the proliferation markers studied, the highest outcome advantage was associated with securin immunopositivity (HR 7.1, *P*=0.0270, 95% CI 1.3–40.1, securin >20 *vs* <10%). In our sample, only securin immunohistochemistry showed statistically significant prognostic value in Kaplan–Meier survival curves (*P*=0.0112) (Figure 3).

## Discussion

Securin is a regulatory protein that plays a central role in DNA repair, p53/TP53 pathway and chromosome stability (Jallepalli *et al*, 2001; Romero *et al*, 2001; Bernal *et al*, 2002). Its expression and localisation are cell cycle-dependent (Yu *et al*, 2000). Human cDNA homologous to rat oncoprotein Pttg was first identified by Dominguez *et al* (1998). It was soon discovered to be highly expressed in several carcinoma cell lines and various human tumours where its abundance was considered as a molecular marker for aggressive disease (Zhang *et al*, 1999). In human tumours, high securin expression has been related to increased cell proliferation and angiogenic phenotype (Kakar, 1998; Ishikawa *et al*, 2001).

Prognostic associations of securin have been reported in gliomas, and in hepatocellular, thyroid and esophageal carcinomas (Shibata *et al*, 2002; Fujii *et al*, 2006; Genkai *et al*, 2006). We have previously described securin expression in a set of genes aberrantly expressed in colorectal carcinoma as compared to paired control samples from normal mucosa (Talvinen *et al*, 2006) where securin expression was upregulated both at mRNA and protein levels. The role of securin in breast carcinoma is not thoroughly studied. Solbach *et al* (2004) published an initial observation on securin mRNA overexpression in association with lymph node involvement and tumour recurrence. In concordance with our present findings, Ogbagabriel *et al* (2005) have reported securin immunohistochemistry in 55 invasive ductal carcinomas and detected a statistically significant correlation with metastatic disease, especially in brain metastases. The paper by Ogbagabriel and co-workers did not analyse associations between securin and other known proliferation markers.

In the cDNA microarray performed, several proliferation-associated genes were markedly deregulated in invasive ductal breast carcinomas (Table 4). According to the magnitude of expression change, the most significantly deregulated proliferation-associated genes were topoisomerase DNA II alpha (TOP2A), securin and insulin-like growth factor 2 (somatomedin A (IGF2)). The prognostic and therapeutic applications of TOP2A and IGF2 have been extensively studied at tissue mRNA and protein levels (Sandri *et al*, 1996; Sachdev and Yee, 2001; Koren *et al*, 2004). The observed significant prognostic value of both TOP2A and IGF2 in our sample supports the reliability of the performed analyses and urges further evaluation of the prognostic value of securin in invasive breast cancer.

Based on our results, securin provides a potential proliferation marker of invasive ductal breast cancer. Comparisons of proliferation markers indicated that securin immunohistochemistry resulted in a different stratification of breast cancer cases, especially identifying patients with a more favourable prognosis than the established proliferation markers MAI and Ki-67. In our sample, a 10-unit increment of securin immunopositivity was associated with a 2.3-fold overall risk of death due to breast cancer (*P*=0.0218) and a 7.1-fold risk for patients with securin immunopositivity above 20% compared to below 10% (*P*=0.0270). Further analyses on larger samples of patients are needed but the present results suggest that securin is a potential proliferation marker that could in clinical pathology add to the information of the traditional prognosticators of invasive ductal breast cancer.

The major shortcomings of the present work are the experimental cDNA microarray methodology and the small sample size with relatively short follow-up time. However, the present results confirm and strengthen the previously published experiences on securin expression in breast cancer (Ogbagabriel *et al*, 2005). To compensate for the small number of patients, we have emphasised the quality of the study material by selecting patients with invasive ductal carcinomas verified by histology, and up-to date diagnostics and follow-up from the era of mammographic screening. In reproducibility analyses between observers and methods, securin performed acceptably although consistencies in evalutions of securin immunohistochemistry were not excellent as for the established methods, MAI and Ki-67. A further point of dispute could be that, in view of recent publications, MAI is optimal in prognostication of node-negative breast cancer of patients under 55 years (Louwman *et al*, 2006; Baak *et al*, 2007). At the moment, we are in the process of expanding our experience on securin immunohistochemistry of invasive and *in situ* carcinomas in a larger sample of patients including menopausal and nodal status.

In conclusion, we introduce a new proliferation marker, securin, which on the basis of biological, immunohistochemical and clinical data is a promising prognosticator in invasive ductal breast cancer along with the traditional proliferation markers.

## Figures and Tables

**Table 1:** Clinicopathological characteristics of patient material in cDNA microarrays and immunohistochemistry (IHC)

	**Microarray (*n*=10)**	**IHC (*n*=44)**
*Age at diagnosis (years)*		
Mean (range)	69 (27–86)	63 (27–87)
		
*Tumour diameter (cm)*		
Mean (s.d.)	3.2 (1.45)	2.6 (1.68)
		
*Histological grade (%)*		
I	20	18
II	30	41
III	50	41
		
*Axillary nodal status (%)*		
Node−	30	54
Node+	70	46
		
*Follow-up time*		
Mean (range)	2 years and 6 months (10 months to 3 years and 5 months)	4 years and 1 month (10 months to 9 years and 11 months)
		
		
*Causes of death during follow-up*		
Breast cancer (%)	20	14
Other (%)	10	7

**Table 2:** Intra- and interobserver reproducibilities of securin and Ki-67 immunohistochemistry, and MAI

	**Securin**		**Ki67**		**MAI**	
	**ICC**	***κ*_w_**	**ICC**	***κ*_w_**	**ICC**	***κ*_w_**
Intraobserver	0.8972	0.7579	0.9577	0.7035	0.9168	0.7632
Interobserver	0.5169	0.3763	0.9286	0.6633	0.9402	0.7484

MAI=mitotic activity index. The interpretations are expressed as intraclass correlation coefficients (ICC) and weighted kappa coefficients (*κ*_w_).

**Table 3:** Univariate analysis of Cox's regression performed on 44 cases of invasive ductal breast cancer for securin and Ki-67 immunohistochemistry, and MAI

	***P***	**95% CI**	**HR**
Securin	0.0218	1.1–4.6	2.3
Ki-67	0.1581	0.9–2.1	1.4
MAI	0.0227	1.0–1.5	1.3

MAI=mitotic activity index. The table summarizes *P*-values with 95% confidence intervals (95% CI) and hazard ratios (HR) of overall breast cancer death.

**Table 4:** Deregulated cell proliferation-related transcripts in invasive ductal breast cancer

**Fold change**	**Type**	**Accession(s)**	**Description**
1.9; 3.6	Up	AA504348; AA026682	Topoisomerase (DNA) II alpha 170 kDa
2.3; 2.5	Down	N54596; N54596	Insulin-like growth factor 2
2.1; 2.5	Up	AA430032; AI362866	Pituitary tumor-transforming 1 (securin)
1.6; 2.2	Down	H28091; R26732	Peripheral myelin protein 22
2.2	Down	T53298	Insulin-like growth factor binding protein 7
2.1	Down	T61948	FBJ murine osteosarcoma viral oncogene homolog B
2.1	Down	H39192	Mitogen-activated protein kinase 7
2.1	Down	AA025819	Growth arrest-specific 1
2.0	Up	T81764	Cell division cycle 27
2.0	Up	AA443982	Protein phosphatase 1, catalytic subunit, alpha isoform
1.9	Up	AA397813	CDC28 protein kinase 2
1.9	Down	AA424584	Latent transforming growth factor beta binding protein 2
1.8	Down	AA455254	Dual specificity phosphatase 6
1.7	Up	AA457710	Polymerase (DNA-directed), delta 4
1.7	Down	AA630376	Notch (Drosophila) homolog 2
1.7	Down	AA633993	CDC10 (cell division cycle 10, S. cerevisiae, homolog)
1.7	Down	R76553	A disintegrin-like and metalloprotease (reprolysin type) with thrombospondin type 1 motif, 1
1.6	Up	T77733	Tubulin, gamma 1
1.6	Down	AA599092	Protein phosphatase 2 (formerly 2A), catalytic subunit, alpha isoform
1.5	Up	AA450265	Proliferating cell nuclear antigen
1.5	Up	AA456077	Sjogren's syndrome/scleroderma autoantigen 1
1.5	Up	N27159	Inhibin, beta A (activin A, activin AB alpha polypeptide)
1.5	Up	AA401479	Cyclin-dependent kinase 5
1.5	Up	W95001	Cell division cycle 25C
1.5	Down	AA428365	Retinoblastoma-binding protein 4
1.5	Down	AA490213	Transducer of ERBB2, 1
1.5	Down	AA490473	Protein phosphatase 2 (formerly 2A), catalytic subunit, beta isoform
1.5	Down	AA456321	Insulin-like growth factor 1
1.4	Up	H20743	Cell division cycle 34
1.4	Down	AA598836	Cullin 4A

The table includes accession numbers, type of expression change and average fold change of analysed samples. When two probes were on array, both accession numbers with corresponding fold changes have been mentioned.
